# The Social Nature of Perceived Illness Representations of Perinatal Depression in Rural Uganda

**DOI:** 10.3390/ijerph15061197

**Published:** 2018-06-07

**Authors:** Nandini D. P. Sarkar, Azucena Bardaji, Koen Peeters Grietens, Joske Bunders-Aelen, Florence Baingana, Bart Criel

**Affiliations:** 1 Health Systems and Equity Unit, Department of Public Health, Institute of Tropical Medicine at Antwerp, Nationalestraat 155, 2000 Antwerp, Belgium; BCriel@itg.be; 2 Athena Institute for Research on Innovation and Communication in Health and Life Sciences, Faculty of Sciences, Vrije Universiteit Amsterdam, De Boelelaan 1085, 1081 HV Amsterdam, The Netherlands; j.g.f.bunders-aelen@vu.nl; 3 ISGlobal, Hospital Clínic—Universitat de Barcelona, Rosselló 132, 08036 Barcelona, Spain; abardaji@clinic.ub.es; 4 Medical Anthropology Unit, Department of Public Health, Institute of Tropical Medicine at Antwerp, Nationalestraat 155, 2000 Antwerp, Belgium; kpeeters@itg.be; 5 School of Public Health, Makerere University, Kampala PO Box 7072, Uganda; kamayonza@gmail.com

**Keywords:** Uganda, perinatal depression, mental health, illness representations, explanatory models, socio-cultural conceptualisations

## Abstract

While the global health community advocates for greater integration of mental health into maternal health agendas, a more robust understanding of perinatal mental health, and its role in providing integrated maternal health care and service delivery, is required. The present study uses the Illness Representation Model, a theoretical cognitive framework for understanding illness conceptualisations, to qualitatively explore multiple stakeholder perspectives on perinatal depression in rural Uganda. A total of 70 in-depth interviews and 9 focus group discussions were conducted with various local health system stakeholders, followed by an emergent thematic analysis using NVivo 11. Local communities perceived perinatal depression as being both the fault of women, and not. It was perceived as having socio-economic and cultural causal factors, in particular, as being partner-related. In these communities, perinatal depression was thought to be a common occurrence, and its negative consequences for women, infants and the community at large were recognised. Coping and help-seeking behaviours prescribed by the participants were also primarily socio-cultural in nature. Placing the dynamics and mechanisms of these local conceptualisations of perinatal depression alongside existing gaps in social and health care systems highlights both the need of, and the opportunities for, growth and prioritisation of integrated perinatal biomedical, mental, and social health programs in resource-constrained settings.

## 1. Introduction

There has been increasing recognition of perinatal mental health, and in particular, of perinatal depression, as a growing public-health concern with various health implications on maternal, infant, and child outcomes [1,2]. Evidence indicates that prevalence rates for common mental disorders are higher in low- and middle-income countries (LMICs) than in high-income countries, affecting up to 15.6% of pregnant women and 19.8% of new mothers in LMICs [3]. In Africa, the prevalence of perinatal depression was found to be 11.3% during pregnancy and 18.3% after birth [4]. There is a small but slowly growing body of evidence focusing on perinatal mental health conditions in Uganda. The prevalence of antenatal depression in post-conflict northern Uganda was recently reported to be 35.8% [5], while postnatal depression has been documented to be 6.1% in a peri-urban setting in central Uganda [6]. Associated factors include young maternal age, unplanned pregnancies, the absence of a stable partner, and marital problems, amongst others [6,7]. However, the likelihood of the progression from antenatal depression to postnatal depression, or the prevalence of puerperal psychosis, remain unknown in Uganda [8]. Similarly, there is a lack of information regarding the impact of perinatal depression or puerperal psychosis on the mother and child in the country [8].

Whether depression, and in particular perinatal depression (PND), is a Western concept, and the extent to which there is cross-cultural equivalence in the illness’ conceptualisations, expressions, and need for treatment, has been an additional debate [9]. It is generally accepted that PND exists in most cultures, and has been reported on in several sub-Saharan countries [10,11,12,13,14]. Nonetheless, to further support the global health community’s continued advocacy for greater integration of mental health into the maternal health agendas [15], a more robust knowledge of the prevalence of PND, and supporting cross-cultural examinations of it, is still required. One way to address this is by understanding local conceptualisations of an illness. Although data exist on the factors related to psychological distress in eastern Uganda [16], on lay concepts of general depression [17], and on the explanatory models of psychotic depression amongst the Buganda in Uganda [18], the specifics relating to PND in Uganda remain unexplored.

### 1.1. Theoretical Framework

The focus of cross-cultural conceptualisations of mental illnesses is usually on culture-specific, word-based expressions, or idioms of distress, which are referred to as *culturally salient indicators of distress* [19,20,21], as well as on the explanatory models of illness, in particular, depression [22,23,24,25]. Illness conceptualisations, however, can be shaped by both the underlying socio-cultural contexts that influence how illness characteristics are perceived, as well as how they are coped with [21,26]. Hence, the Illness Representation Model (IRM) [27,28,29,30] was chosen to explore the dual nature of illness conceptualisations. This model, with its five dimensions of illness representations, was deemed to be the most appropriate for exploring and understanding the nuances within culturally-variable health phenomena, as can be the case with mental health conditions which are often stigmatised and/or without recourse to care in resource constrained settings.

The IRM is a theoretical model from health psychology which is used as a cognitive framework for understanding illness conceptualisations. Its key construct is rooted in the idea that illness representations, or the ‘lay’ beliefs about an illness, are composed of five dimensions [29]. These dimensions are categorised into beliefs regarding: (1) the identity of the illness in terms of symptoms or labels; (2) the causal attributions behind the illness; (3) the timeline of the illness; (4) the consequences of the illness; and (5) illness management in terms of curability, controllability and coherence.

Previous studies using the IRM have largely focused on examining the influence of illness representations on coping behaviours and illness outcomes within the larger Common-Sense Model (CSM) of self-regulation [29,31]. The CSM is a theoretical cognitive framework explicating the processes in which health threats are assessed cognitively and affectively, threat levels are negotiated, and action plans for addressing the health threats are managed, all through continuous feedback loops.

The majority of studies on the IRM have primarily used quantitative factor analytic models focusing on chronic physical illnesses, such as hypertension or diabetes [29]. To the knowledge of the authors, there has been only one study worldwide that has previously used the IRM as a basis for qualitative enquiry regarding conceptualisations of depression [23].

The present study uses the IRM to qualitatively explore multiple stakeholder perspectives regarding illness representations of PND in rural Uganda, by extending the IRM’s usage beyond just the patient’s perspective, towards that of other stakeholders. By situating illness representations through a multi-perspective analysis, socially- and culturally-sensitive narratives of PND in rural Uganda are generated that may enrich our understandings of the phenomena. While this study engages with locally valid conceptualisations of perinatal depression, it stops at framing it within the CSM. Further applications of both the IRM and the CSM may help situate, as well as provide evidence towards, practical and applied improvements in integrated maternal biomedical and mental health service delivery and health care provisions.

### 1.2. Study Objectives

To qualitatively explore multiple stakeholder perspectives regarding illness representations of perinatal depression in rural Uganda using the IRM. It is argued that the IRM can serve as a rich and robust cross-cultural method to qualitatively examine varied acute, cyclical, as well as chronic, mental health problems, such as perinatal depression.

## 2. Materials and Methods 

### 2.1. Research Design and Setting

The outlined study was a qualitative enquiry carried out from March to May 2016 in eastern Uganda as a part of a larger study on the challenge of equitable mental health care at Local Health System (LHS) level in low-resource, rural Ugandan settings (2015–2017). The study setting consisted of four rural villages in Busoga communities, two level-three health centres (HC IIIs) located in the two neighbouring districts of Iganga and Mayuge, and the Iganga district hospital. The study sites were located in the catchment area of the Makerere University Center for Health and Population Research and the Iganga-Mayuge Health and Demographic Surveillance Site.

The HC IIIs are part of Uganda’s decentralised, tiered public health system, and are lower-level frontline health facilities providing basic out-patient, maternal, and infant health services. Iganga district hospital is a general hospital providing preventive and curative outpatient services, maternity services, as well as inpatient, emergency surgery and blood transfusion, and laboratory services. Recent demographic indicators show that the region has a Crude Birth Rate of 30.62, a Total Fertility Rate of 3.4, a Crude Death Rate of 6.4, neonatal mortality of 30.1, and infant mortality of 59.8 [32].

### 2.2. Study Population, Sampling and Recruitment

The study population included a variety of LHS stakeholders from both the community and the informal and formal health care systems (see Table 1). 

Purposive and snowball sampling strategies were utilized to enable maximum variation of identified participants through gradual selection processes. Recruitment at community level was initiated through the Makerere University Center for Health and Population Research and the Iganga-Mayuge Health and Demographic Surveillance Site; the latter institution has been working in the area since 2004. They introduced us to the four village Local Council 1 (LC1), who are the first level of elected local government administrative officials in charge of each village. The LC1 provided contacts for local Village Health Team (VHT) members, who were the main contact points through whom purposive sampling of remaining community members took place, including the perinatal women, their partners, and religious leaders. Recruitment of health professionals was conducted through the local district administrations and district health offices, which provided initial contacts at the district hospital and HCIIIs. This was followed by purposive sampling of health workers, which led to further snowball sampling in field.

### 2.3. Data Collection

In-Depth Interviews (IDIs) and Focus Group Discussions (FGDs) were considered to be the most appropriate methods for generating in-depth information regarding individual and community perceptions regarding PND and its representation. Both IDI and FGDs were guided by the use of a standard case vignette describing the thoughts, feelings, and symptoms of either a pregnant woman or a new mother suffering from PND. A team of three local, Lusoga-speaking data-collectors was trained for six days by the lead author. This included two days of piloting, pre-testing, and refining preliminary case-vignettes of antenatal and postnatal depression, as well as the subsequent supporting preliminary IDI and FGD question-guides (see Table 2) in the neighbouring, demographically and linguistically similar district of Kabira. The case-vignettes and IDI and FGD question-guides were further developed in-field based on concurrent data analysis that took place during the data collection process.

Written informed consent was obtained from all study participants prior to data collection. For the illiterate, verbal and thumbprint consent was acquired in the presence of an eyewitness who also signed in their attendance. All interviews and FGDs were audio-recorded, ranging from 30 min to 120 min. They were conducted in settings appropriate to the participant and data collection method, i.e., ranging from sitting under a distant tree slightly away from the household whilst interviewing a perinatal woman, to local fields and village halls for FGDs, to unoccupied hospital delivery rooms for interviews with health workers.

The three local data-collectors conducted, transcribed, and translated into English all the Lusoga-language IDIs and FGDs. The lead author and one data-collector conducted and transcribed all the English interviews. Data cross-checks corroborating audio-files and transcripts were conducted by the other members of the data collection team.

### 2.4. Data Analysis

The qualitative data analysis was based on both a theory-driven and emergent, partially-inductive thematic coding process. During the data collection period, daily debriefing sessions were held with the whole data collection team to ensure quality control, as well as to conduct the process of field analysis. The debriefing sessions ensured that all emergent data was analysed in an iterative manner, leading to concurrent content and process discussions throughout the whole data collection period. This process of field analysis in the group enabled the lead author to develop manual data-driven codebooks, which facilitated the on-going theory-driven analysis of thematic concerns and trends as they arose whilst in the field. This process also allowed for the emergent realisation of data saturation as it occurred. Once relative data saturation had been reached and the data collection process concluded, all transcripts were individually and manually coded using both theory-driven and data-driven codebooks in the qualitative analysis software NVivo 11 (QSR International, Doncaster, VIC, Australia). The lead author would routinely receive feedback from senior authors based on analytic discussions, which informed the final interpretations as presented here.

### 2.5. Ethical Approval

Individual sub-components of this study, such as translated data collection guides, as well as the larger research on the challenge of equitable mental health care at LHS-level in low-resource, rural Ugandan settings (2015–2017), of which this study is a part, received ethical approvals from the Institutional Review Boards of the Institute of Tropical Medicine in Antwerp, Belgium (No. 997/15), Makerere University, School of Public Health in Kampala, Uganda (No. 299), and the Ugandan National Council for Science and Technology (No. HS 1888). 

## 3. Results

The results present a short description of the sample characteristics, followed by a breakdown of the data through the 5 dimensions of the IRM: (1) perceived identity of PND; (2) perceived causes of PND; (3) perceived timeline and course of PND; (4) perceived consequences of PND; and (5) associated help-seeking behaviours for the perceived illness.

### 3.1. Sample Characteristics

A total of 70 IDIs and 9 FGDs (*N* = 64 participants) were conducted with various groups of LHS stakeholders. A total of 32 IDIs were conducted with 16 pregnant women and 16 new mothers, along with 4 FGDs of mixed groups of pregnant women and new mothers, with 28 FGD participants in total. Of the women having partners (the remaining being ‘single’), 16 IDIs and 2 FGDs (*N* = 16) were conducted with their respective spouses. From the larger community, IDIs were conducted with 4 religious leaders (Muslim imam, Catholic catechist, Protestant and Evangelical pastors), as well as 4 village Local Council 1 (LC1) chairs and vice-chairs, who are the first level of elected local government administrative officials in charge of the village. At the informal health care level, IDIs were conducted with 2 traditional healers and 2 traditional birth attendants, along with 2 FGDs (*N* = 14) with Village Health Team (VHT) members who straddle the intersection between informal and formal health systems. At the formal health system level, 10 IDIs with health professionals included 2 clinical officers from the HC IIIs, and 5 nurses and 2 midwives from the district hospital, as well as the hospital medical superintendent. The health professionals in the FGD consisted of 2 nurses, 3 midwives, and 1 HIV positive ‘mentor mother’ from the district hospital only. Table 2 outlines the participant types and data collection schedule. 

The perinatal women, their partners, other community members, and informal service providers were from Busoga communities, and spoke the local language, Lusoga. The majority of formal health professionals identified as Busoga; those who did not could still speak and communicate in Lusoga, as well as English. 

### 3.2. Perceived Identity of Illness

Participant narratives on the perceived identity of illness focused mostly on various idioms of distress, rather than on specific symptomatic descriptions. This may be linked to the usage of case vignettes of PND, which already provided descriptions of thoughts, feelings, and symptoms. For instance, the majority of stakeholders described the case vignette as depicting a perinatal woman as *“thinking too much”* or *“having too many thoughts”*, and often referred to how this idiom of distress was both a symptom of, and was causally linked to, the perceived identity of the illness. Across the participant groups, the perceived identity of PND fell into three inter-related, yet independent categorizations of illness. The expressed idiom of distress primarily focused on the perinatal woman’s positionality, and was closely related to causal models of explanation, rather than on a symptomatology of illness (see Table 3). 

The first categorization of the perceived identity of PND was that of it *being external to the woman*. The perinatal woman was viewed as a passive recipient of the illness, and the woman was believed to bear no active role in its occurrence and experience. As such, she was seen as being helpless, as suffering, or being confused, i.e., a victim of the illness. 

In contrast, the second PND identity conceptualization placed the illness as *being internal to the woman*. In this instance, the woman was considered to be an active participant in her mental ill-health. There was a connotation of blame and judgement towards the woman, and she was perceived as being foolish, stupid, or even someone who now behaves differently than in the past, and is thereby at fault, i.e., someone responsible for her own condition.

The third category of perceived identity to emerge from the narratives revolved around the perinatal *woman as related to man* in her experience of PND. In this perspective, there was a simultaneous mix of the first two external and internal categorizations, i.e., she could be both a victim of, as well as the responsible party for, her illness, but specifically within the contexts of her positionality to man. Consequently, the perinatal woman with mental health problems was viewed as a woman who was once, but is no longer, loved by someone, or a woman who was left by her partner, or a woman who failed to get married.

### 3.3. Perceived Causes of Illness

*“I think at the community level we (see) social problems, economic ones...”*

—Enrolled midwife 4 _FGD

This quote from a health worker highlights the fact that despite attributing a wide range of causes to PND (see Table 4), for most community members, social and economic factors were the predominant modes explicating women’s mental distress during the perinatal period.

***Partner Related Distress***. Amongst the social factors, partner-related issues were the most frequently cited and strongly perceived cause of PND. These partner-related causal beliefs ranged from *“the lack of love from husband”* to experiences of intimate partner violence and domestic abuse. A commonly cited causal belief was that *“the husband does not provide for the woman”*.

***Characteristics of the Family Unit and the Role of Social Support.*** In addition to the partner related social-support factors, other social causal explanations revolved around the family unit. This ranged from having no parents to turn to for help, to jealous and competitive co-wives, to mistreatment and abuse from the in-laws. A lonely woman with no social support system was frequently perceived as being at greater risk of PND. 

***Supernatural and Spiritual Attacks.*** These social causal beliefs were also intricately interwoven with socio-cultural causal models involving the spiritual and supernatural realm. Two recurrent themes arising from the causal narratives for a perinatal woman’s depression were that of (i) displeased ancestral spirits placing a curse on a woman’s partnership with her husband, or (ii) an *abayiwa* (witchdoctor) bewitching a partnership upon solicitation of co-wives or in-laws.

***Poverty and Lack of Essentials.*** A lack of providing was often linked to other economic causal beliefs of PND being poverty and diet-related, in which the lack of food, or a balanced diet, or basic needs such as bedding, all contributed to a pregnant woman or new mother’s experience of distress and depression. 

***Pregnancy and Perinatal Perceptions***. In a small number of cases, causal beliefs were pregnancy or delivery related. Some individuals thought it a normal situation of the perinatal period, while others thought it was related to fears and stress regarding potential delivery complications. In some cases, women mentioned how their own experiences of PND were linked to the pregnancy being unwanted and/or how often they considered abortion. 

***Medicalisation of Illness***. Medical models of causation for PNDs were linked to having a specific disease, such as HIV, malaria or ulcers (due to the lack of food), or the fear of contracting HIV from their partner. Some participants also quoted PND as being a *“worry related”* illness.

Despite the thematic presentation of causal beliefs, it should be noted that these perceptions of illness causation are very much intertwined as reflected in the following quote:

“For me I think what causes that is that if some men make you pregnant, they neglect you. Some women we get sick and your condition changes from the normal one, we need to eat in a different way but he has neglected you… you don’t want to eat anything so you stay hungry… you are feeling sick but you can’t move to the health facility because he can’t even provide to you transport to go to the health facility. So, you are fed up and you even hate giving birth, and you reach an extent of saying that dying is better because you feel giving birth is useless because you can tell him something but he doesn’t care about you… So, you feel the situation has become hard… for me I think that is what causes that.”

—Partnered pregnant woman 2_FGD2

### 3.4. Perceived Timeline and Course of Illness

Participant perceptions regarding the timeline and course of the PND was the least expressed dimension of illness representation. Nonetheless, PND was generally perceived to be a common occurrence during the perinatal period across the various stakeholders. Throughout the course of the study, multiple perinatal women shared their own experiential understandings of it, as well as informal and formal health workers receiving depressed women:

“It normally happens… and we go through it so much. It is the one I am going through...”

—Partnered pregnant woman 4_IDI

“I have received some (mothers with depression). The woman comes and explains to me that maybe since giving birth, am feeling like this and that, then I give her advice. I have never got one who thinks about death but I get some with a lot of worries and thoughts.”

—Traditional birth attendant 1_IDI

Another illness timeline feature was differences in perceptions between stakeholders regarding the course that PND would take. It was often stated that a woman would recover quickly from PND if she received the appropriate social support and treatment. However, there was a marked difference in the way partners viewed the timeline of the illness and the speed of recovery, as compared to the perinatal women themselves. This may be attributable to the fact that while partners could only try and put themselves in the shoes of the women, in some instances the perinatal women spoke out about their own lived experiences of PND, and, thus, knew first-hand the challenges of recovery and rehabilitation from PND in this particular context:

“For her situation to improve, it needs the previous situation (of lack of provision) to normalize first… It doesn’t even take a week, it’s very easy…”

—Partner of new mother 2_IDI

It will be quite long (to recover), as long as she doesn’t get someone to counsel her on the situation and how she could go about it. The new born could (age up to) two years if she (does not) get counsel from anyone about the situation. If at all she could find someone to counsel her within those 2 years, the period could be less…”

—Partnered new mother 1_IDI

### 3.5. Perceived Consequences of Illness

While this dimension of the IRM did not receive as much attention by the participants as some others, the majority of participants recognised the various corollaries that could arise from a woman suffering from PND. The perceived consequences of a woman suffering from PND fell largely into three categories of recipients: the woman, the new born baby, and/or the community at large. Most participants recognised the possibility of a woman with PND committing self-harm or suicide:

“If the situation continues that way without any remedy, based on her situation she may do harm to herself, she might think of abortion and even committing suicide...”

—Partner of pregnant woman 5_IDI

As the quotes above and below show, the community also expressed concern over the possibility of harm (both intentional or unintentional) that could come to both the foetus and new-born. Abortions and ‘birth defects’, as well as abusive behaviours towards the infants as a result of PND played largely on the minds of the perinatal women and the community:

“If you have many thoughts, the baby will also be thoughtful. She will be spoiling the baby’s brain. The brain will not function very well.”

—Partnered pregnant woman 2_IDI

“It affects my baby, because if I am worried and she is also putting me on pressure as she is crying... and for me who is quick to anger, I slap her...”

—Single new mother 4_FGD3

Lastly, the participants also spoke of various biomedical and social ramifications that could occur not only for the perinatal woman, but also for the immediate family, and how this plays out in the larger community:

“When the community saw her pregnant, they reported (it) to her parents. As you know, the parents they just quarrel and take her for a medical check-up; once they realize that she is pregnant, some parents have a tendency of saying that ‘for me I do not like the person who impregnated you, so let us abort’ and if she refuses, they neglect her, that ‘since she refused, let her suffer’ and other people start laughing at her.”

—VHT 5_FGD2

### 3.6. Associated Help-Seeking Behaviours and Illness Management

This dimension of the IRM was one of the more expressed upon facets of PND’s perceived representation. Participants proffered a variety of strategies, which they believed to be appropriate and adequate towards remedying the primarily social affliction of PND. The majority of these illness management strategies were not necessarily “health” seeking, but rather, were primarily non-health related mechanisms through which the community believed perinatal women’s overall mental health and well-being outcomes could be ameliorated (see Table 5). 

***Partner Related Strategies.*** The most prominent and recurrent strategies involved the partners of the perinatal women. These ranged from behaviours aiming at actively improving the relationship with the husband, leaving/divorcing him, and/or empowering oneself financially and not having to rely upon him for financial support.

***Socially Related Interactions and Behaviours.*** Further modes of improving mental health outcomes revolved around other socially-related interactions and behaviours. Women were prompted to seek advice from their parents, friends, religious leaders, village chiefs, etc. *“Being-open”* and *“not hiding one’s worries”* were perceived as being useful while engaging in greater communication efforts with other community members. Similarly, other socio-behavioural strategies included improving relationships with one’s in-laws and/or co-wives (for those in polygamous relationships). Most accounts of these social strategies with other community members were done in the hope of eventually improving the relationship with the husband, which was believed to be at the crux in improving the woman’s well-being.

***Supernatural and Spiritual Treatments.*** For those instances where PND was perceived to be a result of spiritual attacks or supernatural causes, the first resort was seeking help from traditional healers. Traditional healers were primarily sought to appease any angered ancestors, in order to improve relations with the husband, or prevent bewitchment from co-wives or other jealous community members.

***Biomedical Health-Seeking Behaviours.*** If a perinatal woman’s depression was thought to be biomedical in nature (such as fearing HIV infection), then her associated health-seeking behaviour would be to go to the health facility to receive medication and treatment for the underlying medical condition. Nonetheless, traditional help was often sought as a secondary option once conventional biomedical care was deemed to have failed.

These ways of coping or treatment narratives were not linear, nor individually compartmentalised and segregated. More often than not, the narrated help-seeking behaviours for PND were a complex interplay happening simultaneously and/or sequentially, in some cases in a trial-and-error fashion. The following is an example of this pluralistic approach in treatment and coping:

“The health care provider should give this woman adequate counselling to this woman, and then she can go for the cleansing process to take away all her problems of the family spirits. The health workers should not segregate these women when they return with a changed status after the shrine, the health workers should embrace them and provide the care as appropriate.”

—Partner of pregnant woman 6_IDI

Finally, it can thus be seen that local communities perceived PND as inherently linked to a woman’s positionality, with a predominant focus on socio-economic and cultural causal factors, in particular to partner-related factors. PND was also perceived as being a common occurrence in this context, and it was recognised as having negative consequences for women, infants and the community at large. Related appropriate ways of coping, both individually and as a community, as prescribed by the variety of stakeholders, were also primarily socio-cultural in nature. The following excerpt highlights these mixed nuances:

“Many young girls are reportedly carrying out abortion because care for them is poor or not there at all. Most men in this community have many relationships even when they are not able to provide financially for the many woman they have. Therefore, they end up abandoning the women and hence this causes them to develop many thoughts. This ruins their future. A law must be put in place against early marriage.”

—Village LC1 chair 1_IDI

## 4. Discussion

To the knowledge of the authors, this is the first study exploring grounded conceptualisations of perinatal depression using the Illness Representation Model as a basis for qualitative enquiry in sub-Saharan Africa. In-depth interviews, as well as the usage of case vignettes, elicited in-depth narrative data from a variety of local health system and community-based stakeholders regarding mental health and ill-health during pregnancy and postnatal periods. The IRM allowed for systematic and detailed analyses of the ways in which local Busoga culture viewed and shaped PND illness representations. 

The local representations of the various dimensions of the IRM emphasised the social nature of perceived perinatal depression. A combination of factors attributed to the sociocultural context, and to the woman’s social well-being, played a central role in outlining the identity of PND, associated causal attributions, potential timelines for the illness, its consequences, as well as its management. 

Within these Busoga communities, the identity of PND fell largely within the realm of idioms of distress [19,20], in particular, that of *“thinking too much”* or *“having too many thoughts”.* This is similar to findings of Okello and Ekblad [17] regarding conceptualisations of general depression amongst the Baganda in Uganda. However, supporting the findings of Kaiser et al.’s qualitative review [21], the present study’s findings indicate that this idiom of distress was not merely an expression of symptomology, but rather, simultaneously, also the causal explanation of a perceived social (and in some cases, physical) problem. These causal identities of PND were primarily situated around a woman’s mental and social sense of self, i.e., where the idiom of *“thinking too much”* is either internally attributable to her and viewed as her fault, or external to herself, in which she is more of a victim, or as a consequence of her existence in relation to a man. 

The woman’s positionality in PND is further highlighted and reinforced in the predominantly social nature of the remaining dimensions of IRM. The causal explanatory models voiced across all groups of study participants are distinctly related to social and interpersonal problems of causation, followed by socio-economic and supernatural reasons. While lack of social support and conflicts with partners, in-laws and/or parents are frequently cited causal attributions or known risk factors for general and perinatal depression in LMICs or Africa [3,4,6,7,12,13,17,25,33,34,35], the role that the perinatal woman plays in these conflicts is repeatedly brought up in this study’s narratives. Her social position in relation to others, in particular to her husband or partner, and her own role and responsibility within such a precarious situation, defines how she is perceived by others. Importantly, this social positioning is also perceived to play a role in her own mental health during the perinatal period.

Most participant groups recognised that real consequences can exist for the women, the new-borns, and the community at large; these findings are similar to those from the neighbouring country of Democratic Republic of Congo [10]. However, the extent to which the perceived problem of PND was thought to be curable or preventable was dependant on the stakeholders’ notion of what was wrong, which was especially highlighted in the way in which perinatal women voiced their experiences compared to others.

Coping with PND, conceptualised primarily in terms of social and cultural issues, was centred around activating social support, especially from the partner. This is similar to the findings of previous research on general depression in Uganda [7,17,36]. Most coping strategies were socially and culturally sanctioned, and aimed at improving interpersonal relations through either direct means (such as through open communication with partner) or indirect means (such as through lifting of a curse from co-wife by visiting the traditional healer). Biomedical treatment was therefore often sought in conjunction with traditional health-seeking behaviours, supporting previous traditional healing and health-seeking research in Uganda [16,37]. Strikingly, formal maternal health providers also shared similar conceptualisations with other community members, especially in regards to the causal beliefs of PND. This highlights the need for greater sensitisation and awareness of maternal mental health issues, not just amongst the community, but also within the larger health system, through training for maternal health workers, and service delivery itself.

Situating PND as a socially-constructed illness and social problem in the context of rural Uganda allows for more nuanced understandings and comparisons of perinatal depression as a psychiatric disorder, as compared to a culturally-normative expression of psycho-social, socio-economic, and socio-cultural issues. While there is indeed a sense of distress in the narratives of the perinatal women expressing their own lived experiences in this study, there is need for caution in potentially pathologising and over-medicalising the human expression of sadness and related normative coping behaviours [20,26,38]. As Haroz et al. [39] have shown, the lack of certain globally mentioned features of depression in current gold-standard diagnostic criteria—such as social isolation, loneliness, both of which featured in this study’s results—highlights that both the discussion, and maintaining a critical eye, are still relevant. 

This is not said to compound upon any doubts surrounding the legitimacy of mental ill health and psychological distress; rather, it is intended to support the need to remain critically aware that both an over-medicalised or a primarily socially-constructed perspective of PND may contribute to a moralizing tendency of so-called “normalcy”, and in turn, may create blind spots vis a vis other perspectives. The line between normal and pathological is where a culture and a society chooses to draw it; in this case, this primarily social representation of PND, as offered through the lens of the IRM, may potentially lead to under detection of PND in rural Uganda. Understanding these socially constructed boundary lines of PND can help in informing both mental and maternal health agenda, policies, and services. 

The implications for health policy, practice, and research in Uganda include the fact that, while a national mental health policy exists in the country which includes maternal mental health, there is currently no mental health plan [8]. The lack of dedicated maternal mental health services and guidelines [8], combined with the perceived low quality of interpersonal maternal health care in this setting [40], creates waves of ineffective and unintegrated maternal health care provision towards perinatal women. Further efforts specifically aimed at screening, diagnosing and providing treatment options in such resource-constrained settings need to be initiated. Integrated maternal mental health care as part of primary health care, community-level maternal care, or facility-based care, along with sustainably engaging VHT members, all need to be explored and advocated for from both policy and implementation perspectives.

Additionally, the social dimensions of PND in rural Uganda make the case for a performant social services system which co-exists and coordinates with the health services system, especially at the LHS level. While the health system caters down to the village level, there are hardly any similar decentralized social services. As some of the excerpts demonstrate, most social care for PND is provided on an informal basis by informal caretakers or various community members, such as VHT members, religious leaders, and village chiefs, who provide counselling and advice. The lack of formal, institutionalised social care, and trained social workers at the peripheries of the system, is one aspect which can be improved. Developing the social sector as an adjunct to health systems and their maternal health programs would support the development of people-centred and integrated provisions of care, not just for perinatal women, but also for new-borns and the community at large [41].

Furthermore, while recent reviews have added much to our cross-cultural database of knowledge by highlighting idioms, factors, symptoms, and syndromes associated with general depression and PND [4,21,34,39], it is also imperative to move beyond the perceived illness representations towards actual coping behaviours and illness outcomes. The IRM stands as a good entry point to understanding illness narratives in a culturally sensitive and nuanced manner; however, an added benefit lies in its key role within the Common-Sense Model (CSM) of self-regulation, as proposed by Leventhal [29]. Further research on coping behaviours and illness outcomes through the CSM framework may be useful in this regard.

The strengths of this study lie in the generation of multi-perspective, socially- and culturally-sensitive narratives of PND, which form the basis for developing grounded understandings of the illness in rural Uganda. While the study did not purport to diagnose and identity amongst the perinatal women participants who had PND and those who did not, it is recognised that having complementary diagnostic data could have allowed for a deeper exploration of PND illness representations from the perspectives of women directly affected by it. Additionally, the study itself did not explore the CSM; thus, it leaves open space for future research into understanding more concretely how these illness representations of PND in rural Uganda impact the mechanisms of health seeking behaviours and self-care through individual- and community-level coping behaviours, and how it relays back to actual illness outcomes. Greater measures of control through participant observations and member checking could further validate the findings.

In a real life public health space, the use of the IRM to contextually comprehend PND in rural Uganda through the perspective of various stakeholders potentially allows for the creation of community-based integrated social-health care programs. Informed by the realities on the ground and the primarily social conceptualisations of PND, this study reveals the need for comprehensive and integrated perinatal biomedical, mental, and social health services in these resource-constrained settings. With the ever-growing calls to action for integrating mental health into maternal health care [15,42], further research into the contextualised illness representations of perinatal depression and other perinatal mental health conditions could potentially serve as stepping stones for delineating and working with the real-life complexities associated with improving global maternal morbidity and mortality outcomes. 

## 5. Conclusions

Using the Illness Representation Model as a framework for qualitative enquiry, findings from this study suggest that the perceived illness representations for perinatal depression are largely social in nature, across perinatal women and other stakeholder groups in rural Uganda. Placing these dynamics and mechanisms of local conceptualisations of perinatal depression alongside existing gaps in the social and health care systems, the study highlights opportunities for growth and prioritisation of integrated mental and maternal health interventions and programs that are aimed at improving overall maternal health outcomes.

## Figures and Tables

**Table 1:** Participant types and data collection schedule.

Participant Type	In-Depth Interviews	Focus Group Discussions (Total Number of Participants)
Perinatal women:		
Single	16	2 (12)
Partnered	16	2 (16)
Partners of partnered women	16	2 (16)
Religious Leaders	4	-
Village LC1 chairs and vice-chairs	4	-
Traditional Healers	2	-
Traditional Birth Attendants	2	-
Village Health Team members	-	2 (14)
Formal Health Care Providers	10	1 (6)
Totals	70	9 (64)

**Table 2:** Tentative IDI and FGD question guide.

Tentative question guide based around a Case Vignette describing a pregnant women/new mother suffering from depression. The questions were formulated around the five dimensions of the Illness Representation Model. All questions were open to further probing and exploration.
What do you think is happening to the pregnant woman/new mother? What would people in the community think or say about her? What do people in the community call this? Why you think she is experiencing this? Can you think of what might have caused this? Do you believe that she is suffering an illness? If so, what kind of illness? If not, what could it be? How often do you think women in your community experience this problem? How long do you think this situation will last? Can you think of problems that she could have, because of this situation? How do you think this affects her caring for her unborn/new born child? How should she act upon these feelings? Does she need any kind of help for these feelings? If so, what? If not, why not? If she needs any kind of help, who would be able to help her? What would you advise her to do? Will she feel better with this help? What do you think should be done to give her better help?

**Table 3:** Perceived identity categorizations of PND highlighting a woman’s positionality.

PND Identity Categorizations	Descriptors ^1^	Meanings
External to woman	*Omunaku*	Helpless
	*Kubonabona*	Suffering
	*Okuyawunga/Zonto/Zilogo/Muwuvu*	Confused
	*Kusiruwa*	Condition of lack of self-awareness and self-perception
Internal to woman	*Mudoofu*	Foolish
	*Mussiru*	Stupid
	*Mudenkunu*	Someone who behaves differently from the past, but now it is negative
Woman as related to man	*Mukyaawe*	Someone who was once loved, but this is no longer the case
	*Munobe*	Someone who has been left by her partner
	*Yadiba*	Someone who failed to get married

^1^ In Lusoga language.

**Table 4:** Classification and examples of participants’ perceived causes of PND.

Category	Perceived Cause	Exemplary Quote
Partner related	Lack of love and care Does not provide for woman Abandonment/Paternity issues ^1^	*“The person responsible for her pregnancy has ignored the responsibility to take care of her and so she gets worried of how she would survive…”* —Partner of new mother 6 _FGD1
	Verbal and physical abuse	*“Your husband is abusing you and mistreating you so that also brings you worries.”* —Partnered pregnant woman 3_IDI
	Polygamy/Affairs	*“Our women in the community, most of them hate polygamous families, as soon as anybody hears any rumour that your husband is going in with another lady, there is a tug of war. There the struggle starts.”* —Enrolled midwife 4_FGD
Family related	In-laws mistreatment and abuse	*“She could have caused it by not relating well with her in-laws whom she is living with. It could be that they don’t see eye to eye.”* —Traditional birth attendant 1_IDI
	Lack of parents to turn to	*“If your parents had paid (school) fees and you drop out of school, they abuse you that ‘look at her, she was just thinking of boys’… You thought that you wouldn’t (need your parents) but now you are suffering…”* —Single pregnant woman 5_IDI
Other social support related	Lonely Lack of friends to talk to	*“She may look at her friends (whom) she used to converse with, she fears them (since) her friends are in a good condition… even when her friends try to advise her, she feels she can’t fit among them…”* —Partnered new mother 9_FGD2
Spiritual/Supernatural related	Displeased ancestors	*“The spirits have their own power and authority, it is based on the differences from the families, in some places if a sacrifice is not offered on behalf of the woman, she may never have peace in her marriage.”* —Partner of pregnant woman 1_IDI
	Bewitchment by jealous in-laws/co-wives	*“…It can even be anyone else who bewitched them, not (only) the mother-in-law or sister-in-law but any other person who interfered with their marriage and wants them to (have) conflict and suffer forever …”* —Partnered pregnant woman 1_FGD1
Poverty related	Lack of basic needs	*“It may be caused by poor conditions of living including poor feeding...”* —Village LC1 chair 1_IDI
	Lack of food	*“Sometimes feeding causes problems, she could be interested in eating something, and she won’t get it as desired, and that stresses her.”* —Partner of new mother 3_IDI
Pregnancy/Birth related	Condition of pregnancy Birth/Delivery fears	*“This situation normally happens… when women have got pregnant and after giving birth...”* —Partnered new mother 6_FGD1
	Unwanted pregnancy ^1^	*“Young girls who are 17–19 years, (they have) unplanned or unwanted pregnancies… and no love for the expected baby and then the stigma itself stands that she is young, she is pregnant…”* —Enrolled midwife_IDI
Medical/Disease related	HIV/fear of infection from partner	*“(She may) have got pregnant and she finds out that the person she loved is HIV positive, so she thinks a lot and even hates the baby she has delivered.”* —VHT 7_FGD2
	“Worry” related	*“Of course, she is sick because there is nothing heavier than thoughts. Thoughts are the biggest cause of sickness.”* —Partner of pregnant woman 4_IDI

^1^ Cited more often for and by ‘single’ perinatal women.

**Table 5:** Classification and examples of associated help-seeking behaviours and management for PND.

Category	Exemplary Quote
Partner related	*“She can go and find a job for herself, make her (own) money and in the future, build her own house… the man will just hear about it (since) she leaves the man…”* —Partnered new mother 4_IDI
Socially related	*“If someone has not come to you or you have not learnt about her situation because that person may not be social, then you cannot know what that woman is going through… now if someone has a problem and does not converse with people, then they cannot be helped…”* —Religious leader 3_IDI*“We can tell her to first check (her)self, and we have local marriage courts… we send them there for counselling.”* —Village LC1 chair 4_IDI
Spiritual/Supernatural related	*“The majority (of women, go to traditional healers) because they believe that is the only working God. Going for witchcraft, they give you things - ‘Now you shower with these things, put these in your husband’s food and he will become soft (kind), providing for everything and giving you whatever you want.’ This is being practiced so much in the villages. Actually, women also come to the facilities when they have tied charms around their bodies saying ‘this one is for my husband to love me so much’…”* —Clinical officer 1_IDI
Bio-medically related	*“She should first go to hospital and then to the shrines if they have failed in hospital.”* —Partnered new mother 6_IDI
